# Evaluation of the Association between Fetal Cardiac Disorders with Choroid Plexus Cyst in Fetuses

**DOI:** 10.3390/jcdd9020060

**Published:** 2022-02-16

**Authors:** Mohammad Sedigh Dakkali, Arman Karimi Behnagh, Shakiba Ghasemi Assl, Atiyeh Kimiaeifar, Mohammad Radgoodarzi

**Affiliations:** 1 School of Medicine, Zahedan University of Medical Sciences, Zahedan 9816743463, Iran; dakkali.ms@gmail.com; 2 Echocardiography Research Center, Rajaei Cardiovascular, Medical and Research Center, Iran University of Medical Sciences, Tehran 1995614331, Iran; karimiarman74@gmail.com; 3 School of Medicine, Iran University of Medical Sciences, Tehran 1449614535, Iran; shaakiibaa@gmail.com (S.G.A.); atiekimia1996@gmail.com (A.K.); 4 Department of Pediatrics, School of Medicine, Iran University of Medical Sciences, Tehran 1449614535, Iran

**Keywords:** choroid plexus, congenital heart disease, echocardiography, fetal cardiac abnormality

## Abstract

Choroid plexus cysts (CPCs) are often transient and benign findings observed in pregnancy screenings. This study aimed to examine the association between the frequency of congenital heart diseases and the detection of CPCs. In this prospective case-control study, pregnant mothers with no predisposing risk factors for the development of fetal cardiac abnormalities were eligible for entry. Based on the presence or absence of CPCs on ultrasound, the enrolled fetuses were divided into two groups. All patients (*n* = 100) underwent two-dimensional and color Doppler echocardiography to identify potential cardiac anomalies. Overall, CPCs were detected in 53 enrolled fetuses, and the remainder were enrolled as controls (*n* = 47). Pathological findings, such as echogenic intracardiac focus (EIF), ductal spasm, atrial septal defect (ASD), pericardial effusion, cardiomyopathy, and congenital heart disease were found in neither group. In the CPC group, two mild and six trivial cases of tricuspid regurgitation (TR) were detected. In the controls, five cases of trivial TR were identified. In conclusion, the presence of CPCs was not associated with significant functional or structural fetal cardiac abnormalities, which may be due to altered developmental mechanisms.

## 1. Introduction

Choroid Plexus Cysts (CPCs) refer to small, fluid-filled structures originating from the lateral ventricles of the fetal brain. These cysts result from the accumulation of cerebrospinal fluid (CSF) in the villi and may resolve with increasing gestational age [1]. Most commonly, CPCs are incidentally discovered during second-trimester ultrasound evaluations and are seen in a total of 1–3.6% of all fetuses [2]. These cysts can be single/multiple or unilateral/bilateral and are equally prevalent in males and females [3]. The clinical significance of CPCs remains a matter of debate. While some studies recognize no clinical significance for CPCs [4,5], other investigations have reported an association between the presence of these cysts and chromosomal abnormalities: CPCs are found in 53% of trisomy 18 fetuses on prenatal ultrasound screening [6,7]. This has inadvertently raised the concern regarding the development of CPCs and their probable clinical associations.

The widespread use of ultrasonography (US) for the detection of fetal abnormalities has also resulted in the more detailed documentation of fetal structural variations. Although the majority of these variations may be part of the natural process of fetal development, others were shown to be of pathologic significance [8]. Prenatal ultrasound is performed twice, at weeks 11–14 and 18–24 of gestation. The detection of specified indicators is used to predict anomalies and chromosomal abnormalities [9,10]. These so-called soft markers include choroid plexus cysts, intracardiac foci, nuchal translucency, echogenic bowel, and short humeral length [11].

Assessment of fetal cardiac function is a routine component of pregnancy ultrasound screening [12]. Cardiac diseases are recognized as the predominant cause of death after birth and claim 8 out of every 1000 live births [13,14]. For comparison, cardiac diseases are six- and four-times more common than chromosomal abnormalities and neural tube defects (NTDs), respectively. It is important to note that only 10% of the fetuses with congenital heart disease have identifiable predisposing risk factors and the remainder develop sporadically [13,14]. In a retrospective study, Norton et al. reported a prevalence of choroid plexus cysts in 26% of infants with congenital heart disease and 12% of infants without heart disease. They yielded the correlation between choroid plexus cysts and congenital heart disease [15]. The majority of cardiac disease can be detected between weeks 16–20 of pregnancy via fetal echocardiography [16]. Prenatal diagnosis of cardiac anomalies improves morbidity and mortality, as well as surgical outcomes, in the neonatal period [17,18]. 

Despite the concern regarding the association between the development of CPCs and structural anomalies in affected fetuses, available data are sparse and inconclusive. In addition, and to the best of our knowledge, no previous study has investigated the association between CPCs and the development of fetal cardiac anomalies. To address this issue, we designed and performed this study to evaluate the necessity of fetal heart echocardiography in fetuses with CPCs.

## 2. Materials and Methods

### 2.1. Study Protocol 

This prospective case-control study enrolled pregnant mothers admitted to Rasoul-Akram and Akbarabadi Hospitals (Tehran, Iran) between 31 August 2020 and 21 September 2021. Prior to enrollment and after receiving adequate information on the process and purpose of the study, all eligible participants were required to sign a written informed consent. This study was approved by the Ethics Committee of Iran University of Medical Sciences (IR.IUMS.FMD.REC.1399.687). 

### 2.2. Study Participants

Singleton pregnant mothers of 18–35 years of age and gestational age (GA) of 18–36 weeks were eligible for enrollment. All the mothers referred for echocardiography, due to presence of CPCs, were asked to provide a complete normal profile of screening tests such as serum screening tests and amnio synthesis prior to enrollment. If such documents were not provided by the mothers, they were excluded from the study. Furthermore, fetuses with abnormal US findings in the first trimester or abnormal US findings at the time of enrollment and mothers with a history of fetal cardiac abnormality, congenital heart diseases in first-degree relatives, chronic disease or continuous use of medication, previous abortion(s) or chromosomal abnormality in previous pregnancies were excluded from the study. Eventually, we excluded participants that used assistant reproductive technology to become pregnant; Since it was already demonstrated that such methods could increase the risk of adverse pregnancy outcomes such as CHD [19]. Then, fetuses were divided into two groups: CPC-positive cases and normal controls. Control samples were defined as fetuses without choroid plexus cysts or other abnormalities on ultrasound. Also, in the control group, there were no subjects with a previous history of congenital or hereditary anomalies as well as similar problems in first-degree relatives. Case-control matching was performed based on maternal age, GA, and fetal sex. 

### 2.3. Echocardiography

To minimize anticipation bias, all echocardiographic evaluations were performed by a single specialist using a Phillips Affinity 70C system (Philips Affiniti 70C, Andover, MA, USA) equipped with a multi-frequency C6-2MHz transducer. Apical, long, and short axis, left ventricular outflow tract (LVOT), right ventricular outflow tract (RVOT), sagittal, and three vessels’ views were assessed. In compliance with the guidelines for fetal echocardiography, edited by the Fetal Echocardiography Guidelines Committee, all subjects were evaluated using the M-Mode, two-dimensional and conventional Doppler echocardiography. Left side cardiac output (LCOP) and Right-side cardiac output (RCOP) were calculated in accordance with the formulas below:

LCOP = 0.785 × (aortic valve diameter) 2 × VTI × HR

RCOP = 0.785 × (pulmonic valve diameter) 2 × VTI × HR

Diastolic filling period (DFP) was calculated as the ratio of total diastolic inflow time to the total cardiac cycle duration for both ventricles. The total diastolic filling time was calculated from the beginning of the E-wave to the end of the A-wave. The total cardiac cycle duration was measured from the beginning of any E-wave to the next E-wave’s beginning. 

Doppler echocardiography was used to measure the MPI (Myocardial Performance Index) of the right and left ventricles based on the following: isovolumic contraction time (ICT) + isovolumic relaxation time (IRT) divided by ejection time (ET). Doppler time intervals were measured from the mitral inflow and left ventricular outflow, tricuspid inflow, and right ventricular outflow.

### 2.4. Outcome

Echocardiography was employed to explore anatomical abnormalities and measure cardiac function indices (CFI). In addition to the monitoring of the fetal heart rhythm, valvular function, especially that of the tricuspid valve, was also evaluated.

### 2.5. Statistical Analysis

Quantitative variables are expressed as mean and standard deviation (mean ± SD), and if the variable lacks normal distribution, median and regarded range were implemented. The qualitative data are presented as frequencies and percentages. To assess for normality, the Kolmogorov–Smirnov test was employed. The comparison of normally distributed data was performed using the *t*-test, while the Mann–Whitney U test was used to compare nonparametric data. To check the equality of the variance to perform *t*-test, Levene’s test was used. In the case of qualitative data, the Chi-square test was employed for the purpose of comparison. The *p*-values < 0.05 were regarded as statistically significant. Statistical analysis was performed using SPSS software version 20 for Windows (SPSS Inc., Chicago, IL, USA).

## 3. Results

**Figure 1:**
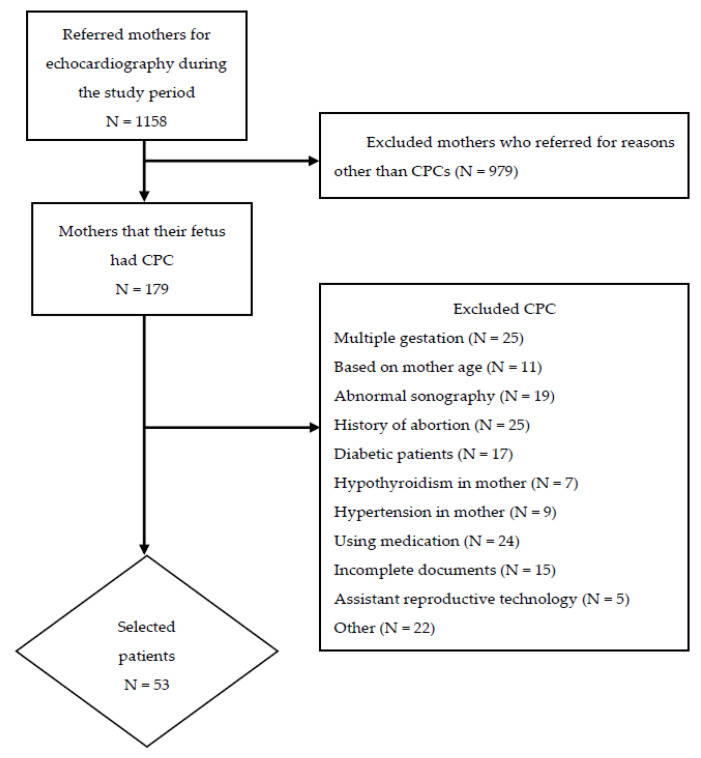
Flow chart of study participants.

In this study, a total of 53 cases and 47 controls were enrolled (Figure 1). In the CPC group, the mean maternal age and GA were 28.06 ± 3.69 years and 25.53 ± 5.65 weeks, respectively, and 47 (88.7%) of the mothers were primigravid. In the control group, the mean maternal age and GA were 27.02 ± 4.26 years and 25.58 ± 5.65 weeks, and 42 (89%) of the mothers were primigravid (Table 1).

The echocardiography results for both groups are represented in Table 2. Echocardiography revealed no structural pathologies, such as echogenic intracardiac focus (EIF), ductal spasm, atrial septal defect (ASD), pericardial effusion, cardiomyopathy, and congenital heart disease in either group. The rhythm was also normal in both groups, and no extrasystole rhythms or atrioventricular block (AVB) were recorded (Table 3). In the case group, a total of six trivial tricuspid regurgitation (TR) and two mild, and in the controls, only five cases of trivial TR were observed (*p*-value = 0.43). 

The echocardiography indices are summarized in Table 2. A mean CT ratio of 0.43 ± 0.01 and 0.43 ± 0.02 was recorded in the cases and controls, respectively. The mean left ventricular ejection fraction (LVEF) was 64.73 and 64.97, while the mean right ventricular ejection fraction (RVEF) 68.94 and 68.47 in cases and controls, respectively. Myocardial perfusion imaging (MPI) seems to be a clinically relevant indicator of cardiac function: in cases and controls, respectively, a mean LV-MPI of 0.43 and 0.51 and RV-MPIs of 0.37 and 0.38 were recorded. No significant difference was observable between the two groups regarding any of the echocardiographic parameters.

Left ventricular posterior wall thickness at diastole (LVPWD), interventricular septum thickness at end-diastole (IVSD), right ventricular wall thickness, and tricuspid annular plane systolic excursion (TAPSE) exhibited non-normal distribution and were assessed using the Mann–Whitney U test. Normally distributed indices were compared using the *t*-test. Overall, only the TAPSE index (*p*-value = 0.036) was found to be statistically different between the two groups.

## 4. Discussion

The current study reported that fetuses with isolated CPCs did not exhibit a higher risk for developing fetal cardiac anomalies. To control for the risk of fetal and congenital anomalies, we enrolled a group of fetuses with isolated CPCs. Moreover, we provided a normal profile of hemodynamic indices in fetuses with CPCs.

CPCs were first observed in the US and described in 1984 and were regarded as normal during development [2]. The formation of CPCs is presumed to be the result of CSF entrapment in the developing choroid plexus [3]. This process initiates in the sixth week of intrauterine pregnancy and culminates in the ninth week with CSF production; thus, these cysts are visible at the end of the first trimester [2].

The correlation between persistent CPCs and various chromosomal abnormalities was suggested. Of these, the association with hydronephrosis and aneuploidies, particularly in trisomy 18 and less in trisomy 21, was investigated more extensively; in addition, an increased frequency of aneuploidy was reported in association with large (> 1 cm) and bilateral CPCs and advanced maternal age (>32 years) [6,7]. The clinical significance of CPCs persisting beyond the second trimester in the absence of other fetal anatomical abnormalities is not evident [4,5].

Nonetheless, Norton et al., who retrospectively examined infants with congenital heart disease, expressed that the prevalence of choroid plexus cysts was 26%, compared to the 12% observed in the controls. However, a noticeable correlation was seen between the presence of CPCs and congenital heart disease and hydronephrosis. Therefore, they proposed non-invasive US screening of the heart and kidney in CPC-positive fetuses [15]. Contrary to their findings, we did not observe a significant correlation between the presence of CPCs and fetal cardiac anomalies.

In our study, the CFI was similar between the two groups, suggesting normal cardiac development despite the presence of CPCs. Overall, the presence of CPCs was not associated with the development of either structural or functional cardiac disorders. This study corroborates previous research that isolated choroid plexus cysts have no clinical significance and are not considered pathological findings.

Tricuspid regurgitation (tricuspid valve insufficiency) may be associated with congenital heart diseases, such as tricuspid valve dysplasia, pulmonary atresia, or atrioventricular canal anomalies or it may be an isolated finding without clinical significance. Messing et al. classified mild tricuspid regurgitation as a benign fetal finding at various stages of pregnancy with an overall prevalence of 83.4% [20]. In a survey conducted by Respondek et al., the prevalence of fetal tricuspid valve regurgitation with normal heart anatomy was 6.8% [21]. Additionally, Gembruch et al. also reported that the prevalence of tricuspid valve regurgitation in normally grown fetuses and those with intrauterine growth retardation is 6.23% [22]. We report a prevalence of 15.09% and 10.63% for tricuspid valve regurgitation in cases and controls, respectively, lacking statistical significance. Furthermore, all cases of tricuspid valve regurgitation were associated with normal cardiac anatomy, congruent with an isolated benign finding [20,21,22].

A major limitation of this study is the limited sample pool; enrollment of larger populations may aid in detecting more minute differences between the cases and controls. A number of analyses, such as investigating the relationship between sizes and numbers and sides of the cysts, were not performable due to lacking data. In addition, the outbreak of COVID-19 completely hampered the postpartum follow-up of the neonates. Nevertheless, and to the best of our knowledge, this study was the first study that focused on the echocardiographic parameters of the fetus heart with CPCs.

## 5. Conclusions

To conclude, the development of CPCs was not associated with structural and functional anomalies. The findings of this study suggest that CPCs, in the absence of previous familial and clinical risk factors, are not associated with the development of fetal heart diseases, and no further screening appears to be necessary for such settings.

## Figures and Tables

**Table 1:** Demographic characteristics of the examined population in the case and control groups.

	Case	Control	*p*-value
Number	53	47	
Mothers’ Age (Mean ± SD)	28.06 ± 3.69	27.02 ± 4.26	0.663
Gestational Age (Mean week ± SD)	25.53 ± 5.65	25.58 ± 5.65	0.94
Number of Pregnancy (Mean ± SD)	1.11 ± 0.32	1.07 ± 0.33	0.213

**Table 2:** Echocardiography indices in case and control groups.

Index (mean ± SD)	Case *n* = 53	Control *n* = 47	*p*-Value
CT ratio	0.43 ± 0.01	0.43 ± 0.02	0.633
LVPWd *	2.0(1.40–3.90)	2.0(1.59–3.40)	0.903
IVSd *	2.1(1.50–4.10)	2.10(1.67–3.70)	0.621
RV wall thickness *	2.16(1.49–4.30)	2.16(1.38–3.90)	0.721
LVEF	64.73 ± 2.41	64.97 ± 1.87	0.582
RVEF	68.94 ± 2.54	68.47 ± 2.63	0.374
LCOP	580.17 ± 95.74	592.8 ± 99.29	0.521
RCOP	600.89 ± 98.09	610.09 ± 98.66	0.644
LV-MPI	0.43 ± 0.02	0.51 ± 0.52	0.273
RV-MPI	0.37 ± 0.02	0.38 ± 0.02	0.063
TAPSE*	0.41(0.38–0.53)	0.41(0.37–0.45)	0.036
MAPSE	0.46 ± 0.02	0.45 ± 0.02	0.50
Mv E/A	0.6 ± 0.01	0.61 ± 0.008	0.302
TV E/A	0.61 ± 0.01	0.61 ± 0.08	0.638
LV-DFP	40.2 ± 0.71	40.46 ± 0.83	0.097
RV-DFP	40.34 ± 0.84	40.42 ± 0.73	0.641
TR velocity	0.75 ± 0.25	0.76 ± 0.09	0.951

CT ratio = cardiothoracic ratio, LVPWd = left ventricular posterior wall dimensions, IVSd = interventricular septum thickness in diastole, RV wall thickness = right ventricular wall thickness, LVEF = left ventricular ejection fraction, RVEF = right ventricular ejection fraction, LCOP = left side cardiac output, RCOP = right-side cardiac output, LV-MPI = left ventricular myocardial performance Index, RV-MPI = right myocardial performance index, TAPSE = tricuspid annular plane systolic excursion, MAPSE = mitral annular plane systolic excursion, MV E/A = mitral valve E-wave A-wave ratio, TV E/A = tricuspid valve E-wave A-wave ratio, LV-DFP = left ventricular diastolic filling period, RV-DFP = right diastolic filling period, TR velocity = tricuspid regurgitation velocity, SD = standard deviation. * These indices were non-parametric and analyzed with Mann–Whitney test and represented as median and range.

**Table 3:** Structural defects in the echocardiography of study participants.

Echocardiography	Case (*n* = 53)	Control (*n* = 47)	*p*-Value
Tricuspid Regurgitation			0.43
Trivial	6	5	
Mild	2	0	
Speed (m/s) (mean ± SD)	0.75 ± 0.25	0.76 ± 0.09	0.951

## Data Availability

Data and statistical analysis are available on reasonable request.
